# Investing in African research training institutions creates sustainable capacity for Africa: the case of the University of the Witwatersrand School of Public Health masters programme in epidemiology and biostatistics

**DOI:** 10.1186/1478-4505-10-11

**Published:** 2012-04-04

**Authors:** Ronel Kellerman, Kerstin Klipstein-Grobusch, Renay Weiner, Steven Wayling, Sharon Fonn

**Affiliations:** 1 School of Public Health, Faculty of Health Sciences, University of the Witwatersrand, Johannesburg, South Africa; 2 Soul City, Institute for Health and Development Communication, Johannesburg, South Africa; 3 Special Programme for Research and Training in Tropical Diseases, WHO, Geneva, Switzerland

## Abstract

**Background:**

Improving health in Africa is a high priority internationally. Inadequate research capacity to produce local, relevant research has been identified as a limitation to improved population health. Increasing attention is being paid to the higher education sector in Africa as a method of addressing this; evidence that such investment is having the desired impact is required. A 1998 3-year investment by the Special Programme for Research and Training in Tropical Diseases (TDR) in research training at the School of Public Health, University of the Witwatersrand, South Africa was reviewed to assess its' impact.

**Methods:**

A descriptive cross-sectional survey of the 70 students registered for the masters programme in epidemiology & biostatistics from 2000-2005 was conducted. Data were collected from self-administered questionnaires.

**Results:**

Sixty percent (42/70) of students responded. At the time of the survey 19% of respondents changed their country of residence after completion of the masters course, 14% migrated within Africa and 5% migrated out of Africa. Approximately half (47%) were employed as researchers and 38% worked in research institutions. Sixty percent reported research output, and four graduates were pursuing PhD studies. Government subsidy to higher education institutions, investments of the University of the Witwatersrand in successful programmes and ongoing bursaries for students to cover tuition fees were important for sustainability.

**Conclusions:**

Investing in African institutions to improve research training capacity resulted in the retention of graduates in Africa in research positions and produced research output. Training programmes can be sustained when national governments invest in higher education and where that funding is judiciously applied. Challenges remain if funding for students bursaries is not available.

## Background

The African region carries a high and disproportionate burden of the world's health problems but finding appropriate solutions to them is complex [1]. One of the contributing factors is a lack of African research capacity to conduct local, relevant research [2,3]. Africa demonstrates an uneven geographical spread of research capacity, and there is a dearth of published research done in Africa for Africa [4]. Only 0.1-0.2% of research articles published in the top 50 biomedical journals between 1995 and 2002 had an African first author [5] and only 1.7 - 7.7% of articles published in the six highest ranking journals on tropical medicine from 2000-2002 were generated exclusively by scientists from countries with a low human development index [6]. Strengthening the research capacity of developing countries is essential to reduce inequities in health, but requires the development of institutional and regulatory frameworks, infrastructure, investment, and a sufficient number of skilled people to conduct and publish research [2,4].

Historically, funding was made available for students to study in developed countries and African students completed their post graduate studies in epidemiology and biostatistics at northern training institutions. Critiques of this have included that it is too expensive, that training is inappropriate to the context in which graduates will eventually work, and that the return rate to Africa is low. Addressing this need for African research capacity has been the concern of international agencies, foundations and research institutions. Some institutions have a long history of investment in building African institutional training capacity and some have entered this space more recently. The UNICEF/UNDP/World Bank/WHO Special Programme for Research and Training in Tropical Diseases (TDR) has over 30 years of experience in various methods of research capacity development in low and middle income countries, such as grants to individuals to pursue higher degrees (often in high income countries), investment in research lead by developing country teams, and awards to institutions strengthening grants usually related to a specific research project [7]. The Partnership for Higher Education in Africa (PHEA), launched in 2000, brought together Carnegie Corporation of New York, the Ford Foundation, John D and Catherine T MacArthur Foundation, and The Rockefeller Foundation for an initial 5 years. PHEA was extended for another 5 years and was joined by the Andrew Mellon Foundation, the William and Flora Hewlett Foundation as well as the Kresge Foundation. Collectively they invested $440 million dollars over the ten years in higher education in nine African countries (Egypt, Kenya, Uganda, Tanzania, Madagascar, Mozambique, South Africa, Nigeria and Ghana). The aim was to develop the next generation of academics. Approximately 84% of the investments went to African grantees and $234 million went in direct support to African higher education institutions. Successes have included building sustained IT capacity, improved infrastructure and increased efficiency [8]. Recent grants by the Wellcome Trust http://www.fic.nih.gov/Programs/Pages/medical-education-africa.aspx and National Institutes of Health http://www.ncbi.nlm.nih.gov/pmc/articles/PMC2623028/ illustrate a growing, ambitious and welcome trend to build African institutional training capacity. There appear to be a number of factors that are of concern to funders: that the investment results in high quality training programmes that train sufficient numbers of students; that the investment accrues to the region as a whole rather than just to one country; and that the programme is sustained after funding is curtailed. There is a paucity of evaluations of such investments in the peer-reviewed literature. Such evidence could support funding agencies that are only now making such investments and who consider investing in Africa risky. This paper describes an investment made by TDR in masters level training in epidemiology and biostatistics at the School of Public Health, University of the Witwatersrand, Johannesburg, South Africa.

We start by describing the way in which TDR invested; thereafter we describe the review we conducted and the results of that review. We then describe briefly the current state of the masters programme to illustrate that it has been sustained and describe the factors that have supported that sustainability. We discuss our findings and conclude with recommendations about investing in African research training institutions.

In 1998 TDR aimed to address African research capacity by investing in three African masters' level programmes in epidemiology and biostatistics. The grant provided three years of seed funding to create the institutional capacity to set up the programmes. As TDR had a long history of funding institutional strengthening in low and middle income countries they did not require a northern partner to be part of the proposal and funding was given directly to the institution itself to manage. The goal of these investments was to establish academic training centres of excellence in Africa to focus on African public health problems. It was envisaged that studying in Africa would offer a cost effective and contextually appropriate environment similar to the research centres to which graduates would return. The School of Public Health, University of the Witwatersrand (WSPH) was selected in a competitive process to offer a masters (MSc) level degree in epidemiology and biostatistics. Funding included student fellowships that continued beyond the initial three-year period. TDR continued with its tested method of awarding student fellowships to staff employed in African institutions. Those institutions had to guarantee continued employment and students were expected to return. At the start of the programme TDR funding allowed us to host a workshop that brought together experts in epidemiology and epidemiology training which allowed us to benchmark our course internationally. For the first three years one staff member from the London School of Hygiene and Tropical Medicine, UK was involved in course review and refinement as an external examiner.

### WSPH masters programme in epidemiology and biostatistics

The WSPH MSc programme commenced in 2000 with an intake of three full time students. The MSc includes course work and a research report (mini thesis). Initially the course was offered either as a one-year full time or two to three-year part time degree, since 2009 as a 18-month full time or three-year part time degree. Core and selective coursework modules cover the theory and application of epidemiology and biostatistics, with particular emphasis on developing country settings and health problems. The MSc includes a field visit to the WSPH Agincourt Health and Demographic Surveillance Site based in a rural sub-district of South Africa where students gain practical, population-based exposure to the application of epidemiology and biostatistics [9]. Quality control is maintained by the external examiner system for the course work and research report, drawing upon both local and international experts in the field. The programme is oversubscribed with about 80 applicants for 15 places each year, class sizes have increased more recently.

In 2006 WSPH in collaboration with TDR conducted an impact evaluation of the programme. The aim of the evaluation was to measure whether the WSPH MSc in epidemiology and biostatistics fulfils its role to develop capacity and excellence in the field of epidemiology; to determine if the course attracted students from the sub Saharan region; to assess if graduates remained in Africa after training; if they were working in health related research and if they considered their training useful and had any research output since qualifying.

The content of the MSc is routinely assessed annually through student and module coordinator feedback and adapted on an on-going basis. This evaluation was conducted to assess the longer term impact that the course has on graduates' contribution to their work environment similar to other such evaluations [10].

## Methods

A descriptive cross sectional design was used to survey all full and part time masters students registered for the epidemiology & biostatistics programme from 2000 to 2005 (n = 70). Self-administered questionnaires were distributed during May and June 2006 at a TDR funded alumni workshop and through email to those who did not attend. Two email reminders were sent to non-responders monthly for 2 months.

The questionnaire used both closed and open-ended questions and collected information on students perceptions of the value of the degree, career mapping, professional and research activities, further academic training and communication with other students. Quantitative data were entered and analysed using Epi-Info 3.2.3. The study was approved by the committee for research on human subjects of the University of Witwatersrand and informed consent was obtained from all study participants. Data were anonymised.

## Results

### Survey population

A total of 70 registered students from 2000-2005 were included in the study, 78 minus 8 who exited or deferred. The majority, 63% were from African countries other than South Africa, 53% were full time students and 29% were female.

The response rate for the survey was 42/70 (60%), with considerably higher participation of full time (78%) compared to part time students (39%). Of the 28 students who did not respond most (89%) were part-time students from the Southern African Development Community (SADC) countries. Tracking the non-respondents confirmed that all of them were still resident in Africa at the time of the survey.

### Survey Respondent's demographic profile

The demographic profile of the survey respondents (n = 42) corresponded reasonably well with the profile of the total group of registered students (Table 1). Sixty six percent (19/29) of full time and 15% (3/13) of part time students were funded by external agencies. Half (53%) of the male students were funded compared to only 32% of female students. The most common funding source was TDR. Further sources included ministries of health, embassies and international funding agencies.

**Table 1:** Demographic characteristics of survey respondents compared to all registered students

	Survey Respondents	All students
	**%**	**%**

Students from countries other than SA	63%	71%

Full time	53%	69%

Female	29%	31%

### Completion rates, research output and continued education of survey respondents

Of the respondents who were due to have completed their research report at the time of the evaluation, 76% of the full time students and only 25% of part time students had submitted their research report or graduated. Completion rates of full-time externally funded (74%) and self-funded (87%) students did not differ significantly and there was no significant association between reported quality of supervision and completion of the research report. (p = 0.313)

Twenty-five (60%) respondents reported some form of research output conceived after starting the masters' programme. Respondents had published 31 articles and presented at 35 conferences.

Nineteen percent (8/42) of respondents had pursued further academic training, which included Field Epidemiology and Laboratory Training (FELT), medical degrees, specialist medical training or a masters in public health. Four (10%) had progressed to a PhD at the time of the survey.

### Migration during or after training

**Figure 1:**
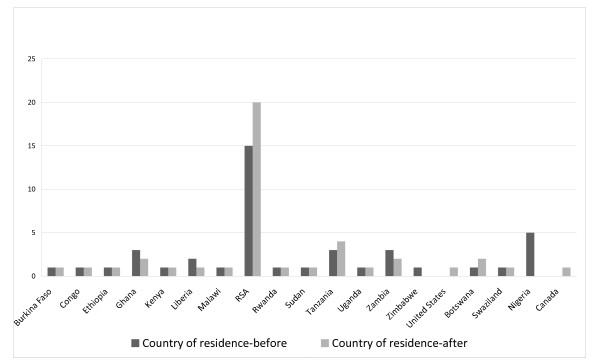
**MSc student's country of residence before enrolling and after completion of the MSc programme in epidemiology and biostatistics**.

Migration patterns before and after completing the course work was documented in the survey. At the time of enrolling in the MSc 14% (6/42) of respondents were resident in African countries other than their country of birth. After completion of the MSc program 19% (8/42) moved country of residence, 14% migrated within Africa and 5% migrated out of Africa. The pattern of movement post-graduation was different for funded compared to self-funded fulltime students; 18% of the funded students compared to 67% of the self-funded students had migrated to other African countries. None of the part time students had migrated. Most out-migrations occurred from Nigeria, whereas South Africa gained a number of new graduates (Figure 1.)

### Application of training

**Figure 2:**
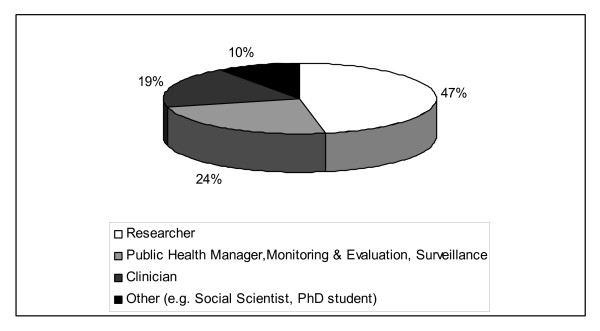
**Job category after completion of the MSc programme in epidemiology and biostatistics**.

**Figure 3:**
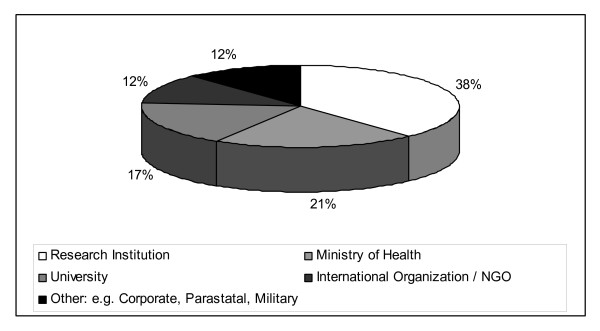
**Work affiliation after completion of the MSc programme in epidemiology and biostatistics**.

To identify if graduates were using the skills gained in the masters programme, we documented the place, type and change in job since completing their course work. Forty-seven percent of respondents are currently working in research positions, with most (38%) working within research institutions (Figures 2 and 3). Of the 50% of respondents who remained in the same job, 14% received a promotion. Forty-three percent of respondents started new jobs, of these 12% moved into a research job and 17% were promoted from junior to senior researchers. Twenty-one percent were employed in senior research management positions and the same proportion as senior public health managers. Fewer funded students changed jobs after completion of the MSc programme. Analysis of the 14 TDR funded students that completed their training 2 or more years before the survey, showed that half of the students (7/14) were still in the same job two years after completing their studies and of those who did change jobs (7/14), did so on average 14 months after completing their studies. Of these, three moved to another African country. Almost 70% of respondents reported that they have trained others in their work environment on epidemiology and biostatistics topics, of which Epi Info, basic statistics and epidemiology were the most common.

### Perceived value of the master's course

Respondents were asked to evaluate the quality and benefit of the course content to their current job on a five point scale. Course modules that were rated high (score > 4/5) were epidemiology, biostatistics, research ethics, infectious disease epidemiology, proposal writing, project management, critical review of articles and doing the research report.

Two thirds of the respondents felt that the MSc programme prepared them adequately for a career in epidemiology. However, further biostatistics and data analysis modules, as well as hands-on research and more data analysis experience were regarded to be important additions to the course to prepare graduates adequately for a research position.

*"I do believe that it (the course) has prepared me by giving me the introductory and basic tools needed. A lot of what I eventually learnt came from hands on experience but it was the initial core training that assisted me greatly and gave me the added advantage over those having not done the MSc"*. (Full time student enrolled in 2003) One student also reported that "*I can now compete very well with epidemiologists who graduated from other countries*."

Respondents felt that the MSc programme could be improved by increasing the course duration to 18 - 24 months, with a strong emphasis on building practical skills (field work and/or field placement for the research project). Project management and grant writing skills were considered core skills to be included as compulsory rather than selective modules. Survey respondents suggested additional advanced modules in epidemiology and biostatistics, additional training in statistical software packages, management or leadership courses, health care financing, and monitoring and evaluation.

Students were asked to comment on the type of services or support they would like the MSc programme to offer post graduation. They expressed a strong demand for a web portal to interact with both staff and students, to provide an active discussion forum for scientific issues including a statistical helpdesk function, access to scientific literature, information on courses, research funding, internships, links to PhD programmes and career opportunities.

### Communication with other students

Networking is seen as an important aspect of developing research partnerships and maintaining a professional community. Respondents were questioned about the frequency and reason for communication with their fellow students. Eighty-three percent of full time respondents were in communication with their fellow students, but only 33% of part timers. The majority of the contact is done through email (59%) followed by telephone (21%) and in person (18%). The reason for maintaining contact was split between social (45%) and work/research related issues (36%).

### Sustainability

The initial 3-year TDR funding allowed the School of Public Health to employ a fulltime academic to coordinate the course and steer its development, fund curriculum development (workshops, visits from international experts), buy books and software, and set up a computer laboratory. Teaching was anchored by the academic coordinator and shared between existing staff. Thereafter an existing staff member coordinated the programme. Student fellowships continued beyond the three years.

The higher education sector in South Africa is supported by a government that allocates government subsidy to universities based on their student numbers as well as their academic output which includes the number of post graduates and publications. With this as a basis, the school lobbied the faculty and convinced them that this new programme brought in new students and thus revenue. The faculty allocated the school funding for additional posts. From no funded posts for epidemiology training in 1989 the school in 2006, when this survey was conducted, had secured three additional full time permanent academic posts. Currently (2011) the university funds seven full-time staff members in the epidemiology and biostatistics division. The TDR funded programme continues and is now in its 11^th ^year with 145 students enrolled over that time. The strength of the TDR funded programme has allowed us to compete for other programme development funding which has increased the areas of specialisation (fields of study) that we offer (epidemiology and biostatistics, population based field epidemiology, infectious disease epidemiology) and extended our training to PhD level [11]. Various short courses are offered and a new field of study in research data management is currently being developed. To date 218 students from some 20 African countries have enrolled at WSPH for MSc degrees in epidemiology and biostatistics (all fields of study). A recent review of the field of study in population based epidemiology showed a 100% completion rate. In order to develop these additional fields and offer short courses it has, however, been essential to apply for additional grants. Expansion without additional investment would not have been possible.

## Discussion

There are very few case studies on the impact of investment in African programmes to build research capacity [2,9,12,13]. This evaluation, while having limitations, does offer some useful information in the peer reviewed public domain. Limitations include the relatively short follow up time. Students may still move out of Africa, thus research output, PhD progression rates and career advancement consequent on skills gained could be underestimated. Including students who had not yet graduated could further exacerbate this underestimation. Comparative information on similar programs is also lacking. Notwithstanding, this evaluation provides valuable insight and lessons on building African research capacity in Africa for Africa.

Capacity building requires longer term investment and at a level significant enough to ensure ultimate sustainability and to provide a critical mass [14]. It is therefore difficult to invest in too many places. The challenge of investing in a limited number of institutions is that the benefits may accrue to local or national players only. The TDR investment specifically intended building regional resources. The evaluation of the Wits School of Public Health programme indicated that just over 60% of the students came from countries other than South Africa. This trend continues today. The other challenge of training people outside of their own country is that they may not return home. A 2003 study showed that only 40% of PhD students returned to their country of origin after studying in the USA [15]. In the WSHP evaluation only 5% of the graduates had migrated out of Africa. Students in Africa do migrate within the continent and those in this programme had moved both prior to enrolling (14%) and after graduating (14%). Movement of scholars within Africa has been described as a sign of solidarity, cooperation and collaboration amongst underdeveloped countries, a collective effort towards socio-economic development [15]. The need for, and scarcity of, research expertise in higher education institutions thus facilitates that African scholars tend to work across borders, which is not always perceived positively as some countries have gained to the detriment of others [16]. This masters level training of Africans in Africa appears to be beneficial to the region.

To contribute to research, graduates need to be working in positions where they can use their skills. Just fewer than half the respondents (46%) described themselves as researchers and a further 23% said they were involved in research related activities such as monitoring and evaluation and surveillance. Another measure of contribution to research capacity is research output. Although this is a relatively young programme and publication output may reasonably be expected to grow with time, there were impressive results with sixty percent of respondents with only a masters degree reporting some form of research output (publication or conference presentation). This also indicates that graduates see the importance of and have the capacity to make their research findings available in the public domain. It implies that the programme has given them sufficient motivation and skills to achieve this level of output. Funders should consider whether it would be beneficial to give graduates start up money for a research project to enhance rapid re-integration into their organisations after they graduate. These re-entry grants are a feature of some programmes and they may be beneficial at masters level too [2]. Pursuing higher degrees is yet another measure of building research capacity and twenty percent of students had gone on to further post graduate training and ten percent to PhD level training. Graduates were also promoted from junior to senior researchers and into research management roles implying a positive contribution of this training to developing research capacity in African institutions. Graduates reported being in contact with each other post graduation. Facilitating this via a research portal would allow them to develop an alumni network across the continent that may be useful in developing research collaborations and developing common protocols for multi-country studies [17].

A large proportion (42%) of respondents are involved in research or public health management, this is a relatively senior position. This rapid career progression might be a reflection of the dearth of professionals trained in epidemiology and biostatistics in Africa placing high demands on masters graduates. To be able to function in these positions, additional skills including project management, proposal, grant writing and financial management seems to be needed as reflected by the survey respondents' comments on how to improve the course. It is not clear that a single master's degree can achieve all of this in the time usually allocated to this level degree programme.

Almost all students complete the coursework on time and pass, completing the research report is a rate limiting step towards graduating with full time students graduating much more quickly than part-timers. While full-time study takes students away from their jobs they do graduate more quickly. This evidence suggests that funding and work place policies that allow for full-time study will advance the number and capacity of African researchers more rapidly. The funding provided by TDR linked the student with their workplace, a mutual obligation to return and to have a place to reemploy the student. TDR-funded graduates did return and the 50% who eventually moved on stayed for an average period at least as long as the period they were away studying.

Sustainability is a central concern of funders. The TDR modest start-up of a $300 000 three-year funding was critical to the success of the programme. It supported the programme through the initial set-up phase when the student numbers were still low. It allowed us to develop a strong curriculum and provided funding for expert external evaluation of the programme which enabled us to benchmark the degree internationally. Continued funding of scholarships for students is essential to sustain the diverse pool of students with their unique experiences and subsequent Africa-wide networking links. It also allows for full-time study. The sustainability of the programme has been facilitated by continued access to student bursaries but these are insufficient and in every year a few students who are accepted do not take up their positions as they cannot find funding.

The importance of government investment in higher education cannot be underestimated - this was an essential element in arguing for permanent posts. However not all African governments invest or invest sufficiently or reliably in higher education.

## Conclusion

The evaluation has shown that investing in African training institutions has provided a regional training resource whose graduates stay in Africa after graduation. A good proportion of the graduates work in research positions, have contributed research output and have gone on to further higher degrees. The programme has not only been sustained but has also developed and over a ten year period more than 200 students have been admitted. Government investment in higher education and the judicious application of those resources is central to sustainability. Challenges that need to be addressed include: the need to secure ongoing scholarship funding; and promoting ongoing learning and networking through innovative resources such as a portal to support advanced distance learning and to promote professional networks and partnerships. The future of Africa's public health depends on building strong African capacity and institutions.

## Abbreviations

CARTA: Consortium for Advanced Research Training in Africa; DSS: Demographic Surveillance Site; FELT: Field Epidemiology and Laboratory training; INDEPTH: International Network for the Demographic Evaluation of Populations and their Health in Developing Countries; LSHTM: London School of Hygiene and Tropical Medicine; PHEA: Partnership for Higher Education in Africa; SADC: Southern African Development Community; TDR: Special Programme for Research and Training in Tropical Diseases; UNICEF: United Nation's Children Fund; UNDP: United Nations Development Programme; WHO: World Health Organization; WSPH: School of Public Health: University of the Witwatersrand.

## Competing interests

The authors declare that they have no competing interests.

## Authors' contributions

RK, SF and SW were involved in the design of the research and data collection instrument, RK conducted the research, did the initial analysis and wrote the first draft of the article. RW, KK-G and SF commented on the analysis and interpretation of the data. All authors were involved in the review of the article and the final version of the paper submitted. All authors read and approved the final manuscript.
